# Effects of a Change from an Indoor-Based Total Mixed Ration to a Rotational Pasture System Combined with a Moderate Concentrate Feed Supply on the Health and Performance of Dairy Cows

**DOI:** 10.3390/ani8100169

**Published:** 2018-10-03

**Authors:** Julia Hartwiger, Melanie Schären, Ursula Gerhards, Liane Hüther, Jana Frahm, Dirk von Soosten, Jeanette Klüß, Martin Bachmann, Annette Zeyner, Ulrich Meyer, Johannes Isselstein, Gerhard Breves, Sven Dänicke

**Affiliations:** 1 Institute of Animal Nutrition, Friedrich-Loeffler-Institut, Federal Research Institute for Animal Health, Bundesallee 37, 38116 Braunschweig, Germany; Julia.Hartwiger@fli.de (J.H.); Melanie.Schaeren@uni-leipzig.de (M.S.); U.gerhards@gmx.de (U.G.); Liane.Huether@fli.de (L.H.); Jana.Frahm@fli.de (J.F.); Dirk.von_Soosten@fli.de (D.v.S.); Jeannette.Kluess@fli.de (J.K.); Sven.Daenicke@fli.de (S.D.); 2 Institute of Agricultural and Nutritional Sciences, Martin-Luther-University Halle-Wittenberg, Theodor-Lieser-Str. 11, 06120 Halle (Saale), Germany; Martin.Bachmann@landw.uni-halle.de (M.B.); Annette.Zeyner@landw.uni-halle.de (A.Z.); 3 Department of Crop Sciences, Grassland Science, Georg-August University, Von-Siebold-Str. 8, 37075 Göttingen, Germany; Jissels@gwdg.de; 4 Institute for Physiology, University of Veterinary Medicine Hannover, Foundation, Bischofsholer Damm 15, 30173 Hannover, Germany; Gerhard.Breves@tiho-hannover.de; 5 Clinic for Ruminants and Swine, Faculty of Veterinary Medicine, University of Leipzig, An den Tierkliniken 11, 04103 Leipzig, Germany; Melanie.Schaeren@uni-leipzig.de

**Keywords:** dairy cows, ration change, pasture, confinement, animal behavior, fat depots, liver fat, rotational grazing

## Abstract

**Simple Summary:**

In grazing systems of temperate climate zones dairy cows are often fed with a silage- and concentrate-based diet during winter and are gradually introduced to a pasture-based diet in spring. This housing and feeding change involves many complex nutritional, behavioral, and metabolic adaptions, likely connected with alterations in energy metabolism. In a previous trial, feeding low amounts of concentrate during full-time grazing had not prevented energy shortage during the first weeks on a pasture system. Because of this, further research was needed to determine whether a higher concentrate supply during full-time grazing would have attenuated that energy deficit. In this experiment we were able to show that the transition period is connected with a higher activity due to walking and grazing, associated with changes in red blood cell count. Further parameters like lipomobilization, decreasing milk production, and loss in bodyweight confirm the alterations in energy metabolism. In summary, the present study shows the changes and its impact on the animals during transition to full-time grazing.

**Abstract:**

In spring, the transition from a total mixed ration (TMR) to pasture requires metabolic adaptions for the cow. It had been shown that supply of low amounts of concentrate after transition to full-time grazing caused energy deficits, resulting in a lower milking performance and changes in a variety of variables indicative for energy metabolism. The present study aimed to investigate how a moderate concentrate supply (4.5 kg dry matter cow/day) after transition to pasture influences health and production indicators. Over a 12-week trial period dairy cows were observed during transition from confinement to pasture (pasture group: PG) and compared to cows fed TMR indoors (confinement group: CG). On average, the PG consumed less feed and energy than the CG and mobilized body reserves, which is mirrored in a decrease of body condition and various fat depots. These effects were paralleled by elevated serum concentrations of non-esterified fatty acids and ketone bodies as well as an increase in liver fat content. The physical activity (elevated walking, eating, decreasing rumination time) of the PG was significantly higher than that of the CG, which intensified the energy deficiency and resulted in a lower milk yield. In conclusion, the moderate concentrate supply was insufficient to counterbalance the lower energy intake from pasture during transition.

## 1. Introduction

In pasture-based dairy production systems, dairy cows are often fed with an indoor-based total mixed ration (TMR) during wintertime and are then introduced to pasture in spring. Many full-time grazing studies deal with topics referring to dry matter intake (DMI) on pasture with or without concentrate supply [1], animal behavior and intake rate [2], or different pasture heights to improve DMI [3]. Aim of these studies was providing the animals with sufficient energy to achieve high milk response during full-time grazing. When it is their only feed source, pasture is the first limiting factor to meet the animals’ energy requirement, particularly for high producing cows [2]. In most of the actual studies, the transition period was not examined or the experimental set up noted that the animals had grazed some days on pasture before the sampling started [2,4]. However, the time needed for adaption and the impact of ration change on the animal has not been extensively researched so far. 

Actual results of the study of Schären et al. [5] examined transition of high yielding Holstein cows in mid-lactation during springtime from a TMR based indoor system to a continuous grazing system combined with 1.75 kg DM cow/day concentrate supply (92 ± 1.1 g DM concentrate/kg milk). Changes in variables indicative for energy metabolism were observed during transition and in a full-grazing system hinting at an unbalanced energy supply, an energy deficiency, a compromised milking performance, and adaption of rumen microbiota. A negative energy balance (NEB) and lipolysis ultimately appear when the energy expenditure exceeds the energy intake due to different reasons. Excessive lipolysis is characterized by a decrease in body condition and a partial loss of fat depots, as well as increased serum concentrations of non-esterified fatty acids (NEFA) and ketone bodies (e.g., beta-hydroxy-butyrate (BHB)). Circumstances with NEB are well-known from calving and mostly associated with great liver fat accumulation [6,7].

On pasture, a lower DMI (61%), and also, additional energy requirements for grazing and walking (24%), an excess of nitrogen excretion with feces (12%), and a higher energy need for milk production (7%), altogether lead to a higher energy expenditure [8]. Besides the higher energy expenditure for walking, such increased outdoor activities might particularly influence the red blood cell count and general health of the cow [9].

To face the mentioned limitations of energy supply during transition, three nutritional factors can be manipulated to influence energy intake; these are herbage availability, feeding value of the offered herbage, and the level of concentrate supplementation. A rotational grazing system with a high sward height and high frequency of paddock rotation can lead to a higher DMI [10].

In sum, the aim of the present experiment was to examine the efficacy of a moderate concentrate feed supply under a rotational grazing system in order to facilitate the adaptation process and alleviate energy deficiency during adaption.

## 2. Materials and Methods

Experimental work was carried out at the experimental station of the Institute of Animal Nutrition, Friedrich-Loeffler-Institut (FLI) in Braunschweig, Germany. The experiment was carried out in accordance with the German Animal Welfare Act approved by the Lower Saxony State Office for Consumer Protection and Food Safety (LAVES), Germany and was supported by the Ministry of Science and Culture of Lower Saxony (MWK), Germany.

### 2.1. Experimental Design and Treatments

Fifty-seven primi- and multiparous German Holstein cows, managed in a seasonally calving pattern (163 ± 32 days in milk (DIM); mean ± SD), were randomly assigned to either a pasture group (PG; *n* = 26) or a confinement group (CG; *n* = 31). Both groups included rumen- and duodenum-fistulated animals (PG, *n* = 6; CG, *n* = 5). Cows already had gained pasture experience before their first calving and during dry periods in previous seasons. At the beginning of the trial both groups were characterized by the main criteria parity (2.1 ± 1.3), milk production (28.8 ± 0.6 kg milk/cow/day), body weight (BW; 618 ± 7 kg), and body condition score (BCS; 2.94 ± 0.01; 5-point scale) according to Edmonson et al. [11]. The experimental work lasted 12-weeks from 18 April to 8 July, 2016. During the weeks before this trial started, all animals had received a partial mixed ration and concentrate feed (available at an automatic feeding station) according to their actual energy and nutrient requirements. Our experimental setup was similar to Schären et al. [5], except for the grazing system (rotational vs. stationary) and the amount of concentrate supply during full-time pasturing. The CG stayed on the same TMR-based diet during the whole trial whereas the PG was slowly switched to a pasture-based ration: week 0 and 1 = TMR, week 2 = TMR and 3 h pasture/day, week 3 and 4 = TMR and 12 h pasture/day, and week 5 to 11 = pasture combined with 4.5 kg DM concentrate/cow/day. The TMR consisted of 35% corn silage, 35% grass silage, and 30% concentrate. Ingredients and chemical composition of the two diets are shown in Table 1 and Table 2. The confinement animals were fed daily at 1100 am. In contrast to the experiment of Schären et al. [5], the PG received from week 5 to 11 after morning and evening milking 4.5 kg DM concentrate cow/day (vs. 1.75 kg DM concentrate cow/day), divided into two equal portions. From week 5 to 11 animals spent approximately 2 h daily in confinement for milking and concentrate feeding.

### 2.2. Pasture Management

In the present trial, a rotational grazing system was performed. Preparing of the pasture started in the transition period winter to spring (fertilization, rolled with a cylinder). A few days before the cows entered the first paddock young cattle had grazed on it. The total grazing area included 6.5 ha without tree vegetation and was divided into four paddocks of 1.6 ± 0.3 ha each. The walking distance between the stable and the most distant paddock was 400 m. The characterization of the pasture (plant species and coverage) was documented in week 2. The pasture height was measured on a daily basis with an electronic rising plate meter (RPM F400, Farmworks System Ltd., Manawatu-Wanganui, New Zealand). All cows entered a paddock when sward height reached an average of 14 cm, and left the paddock when the sward height was around 8 cm or when the visual assessment of the pasture indicated an insufficient composition. In addition, the pasture allowance per animal and hectare was calculated two times weekly by cutting five grass locations above ground level with a frame of 0.25 cm^2^ size, which then were weighed and oven dried. The stocking density for all paddocks was 17 animals/ha. From week 5 to 11 the herd grazed on each paddock for approximately 6 days on average. After each rotation, the paddock was cut and the grass removed. Afterwards, the area was fertilized with calcium ammonium nitrate, manured, and watered regularly.

### 2.3. Weather and Barn Climate Conditions

For the pasture conditions, daily average as well as minima and maxima temperatures were recorded in collaboration with the adjacent Germany’s National Meteorological Service (Deutscher Wetterdienst, DWD). In the confinement system climate data logger (Tinytag Plus 2 TGP-4500; Gemini Data Loggers, Chichester, UK) were applied. The data were used to calculate the temperature humidity index (THI) as described in Hahn [11].

### 2.4. Pasture and Feed Measurements

Representative TMR and concentrate feed samples were collected on a daily basis and pooled over 6-week periods, except for the concentrates for the PG which were collected representatively during the manufacturing process. Pasture samples were collected daily during morning milking and pooled weekly. The samples were taken with the help of an electronic scissor (Gardol^®^ GGSI 180, Münster, Germany) in areas were the cows spent most time for grazing and exclusively from the upper half of the plant. TMR, grass, and concentrate were analyzed for dry matter (DM), ash, crude protein (CP), starch, sugar, ether extract (EE), crude fiber (CF), neutral detergent fiber (NDF), and acid detergent fiber (ADF), the two last measured variables are expressed without residual ash. Utilizable CP (uCP), ruminal nitrogen balance (RNB), and net energy lactation (NEL) of silage and grass samples were determined by near infrared spectroscopy. All methods are based on the procedures recommended by Verband Deutscher Landwirtschaftlicher Untersuchungs- und Forschungs­anstalten [12], as described in details by Schären et al. [5].

### 2.5. Animal Measurements

#### 2.5.1. DMI

In the CG individual DMI and water intake was recorded automatically using electronic weighting troughs with ear tag detection (computerized feeding station, Insentec Typ RIC, B. CF., Markenesse, The Netherlands). To estimate individual DMI of the PG three methods were used; namely the n-alkane method, exclosure cages, as well as a calculation method according to Heublein et al. [13].

The n-alkane method we used was based on Elwert et al. [14]. From week 5 until week 8 each cow of the PG received, after milking, a concentrate/alkane mixture (4.5 kg DM concentrate/day) in two equal portions in order to retrace the DMI on the first weeks of full-time grazing. The DMI on pasture was recalculated as described by Taweel et al. [15] and Dove and Mayes [16] with the help of the concentrate/alkane mixture, particularly the alkane C32 (n-dotriacontane, Acros organics, Geel, Belgium, CAS No 544-85-4). Details of the methodology are presented in Schären et al. [5]. Briefly, the C32 was mixed with cellulose powder (1:10) and added to the concentrate feed before pelleting. Alkane concentration of concentrate feed amounted to 923 mg/kg DM and corresponded to a total alkane dosage of 4.2 g/cow/day at a daily concentrate feed allowance of 4.5 kg DM. Pasture samples were collected once in the morning (0500 h) and feces twice daily after milking. Samples were pooled weekly for every cow. They were stored at −20 °C, freeze-dried, and ground through a 1 mm sieve before alkane extraction, purification, and gas chromatography analysis (Shimadzu GC-2010 (Shimadzu Corporation, Kyoto, Japan) with flame ionization detector (315 °C), and an Rtx^®^-1 w/Integra-Guard™-column (30 m × 0.53 mm, 0.25 µm film thickness; Restek Corporation, Bellefonte, PA, USA). 

Furthermore, the DMI on pasture was estimated using exclosure cages (each 2.75 m^2^), also as described in Schären et al. [5]. Before the PG entered a paddock, next to the future location of the exclosure cage a reference cut of the size of the exclosure cage was collected. Every time the animals were moved to another paddock, the herbage under the exclosure cage was harvested as well as another area of the same size next to it. DMI could then be estimated through the difference in DM content. The paddocks were mostly changed in the middle of the week. Therefore, the DMI of the exclosure cages was calculated from week 5 to 6.5 and so on. Water was available for ad libitum intake at each paddock, but could not be quantified.

#### 2.5.2. Milk, BCS and BW 

Milk yield was measured twice daily (0530 and 1430 h) using automatic milk counters (Lemmer Fullwood GmbH, Lohmar, Germany) and BW was documented automatically after milking. Samples of milk were taken twice per week and stored at 4 °C until analyzed for fat, protein, lactose urea, and SCC concentrations using an infrared milk analyzer (Milkoscan FT 6000 combined with a Fossomatic 5000; Foss Electric, HillerØd, Denmark). The BCS was documented weekly using a 5-point scale according to Edmonson et al. [17].

#### 2.5.3. Blood 

Blood samples of 43 animals (PG: *n* = 22, CG: *n* = 21) were collected weekly on Mondays by puncturing the vena jugularis externa. EDTA blood samples were directly used for hematology, while heparinized plasma and serum samples were centrifuged at 2123 × *g* for 15 min at 15 °C (Heareus Verfuge^®^ 3.0 R Heareus, Osterode, Germany) and frozen in portions either at −20 or −80°C. Weekly, a complete blood count was performed (Celltac α MEK-6450, Nihon Kohden Corporation, Tokyo, Japan), using EDTA blood samples, including hemoglobin, hematocrit, and red and white blood cell counts. Furthermore, weekly serum samples were analyzed for concentrations of BHB, FA, urea, and glucose by using an automatic analyzing system based on photometric measurements (Eurolyser, Type VET CCA, Salzburg, Austria). 

The following blood variables were recorded three times during the trial (week 0, week 6, week 11) serum albumin, total protein, cholesterol, aspartate transaminase (AST), γ-glutamyltransferase (γ-GT), total bilirubin, glutamate dehydrogenase (GLDH), and total triglyceride values; also calculated based on photometric measurements.

#### 2.5.4. Fat Depots

Fat depot mass of 32 animals (*n* = 16) was estimated quantitatively via ultrasonography according to Raschka et al. [18] in week 0 and 11 during the trial. Briefly, a Mindray M5 Vet (Mindray, Shenzhen, China) ultrasound machine equipped with a 6 MHz linear probe (Mindray, 6LE5Vs) and a 3 MHz convex probe (Mindray, 3C5s) was used for transcutaneous ultrasonography. Different definite locations at the right side of the cow were used to estimate the subcutaneous adipose tissue (SCAT), retroperitoneal adipose tissue (RPAT), omental adipose tissue (OMAT), and mesenteric adipose tissue (MAT). The hair around each location was clipped, and conductive gel was applied to improve the bond between the skin and the transducer. The thickness of the tissues of different locations was measured on frozen images. Corresponding to Raschka et al. [18], the equations of the multiple linear regression analyses of the fat depots were used for determination.

#### 2.5.5. Liver Fat

Liver biopsy samples of approximately 100 mg were taken in week 0, 6, and 11 by using an automated spring-loaded biopsy device (Bard Magnum^®^, Bard, Tempe, AZ, USA) with a 12-gauge needle (Magnum biopsy needle 12G × 200 mm, PZN 3405134, Bard Tempe AZ, USA) under local anesthesia (procaine hydrochloride; Isocaine 2%, Selectavet, Weyarn-Holzolling, Germany). Directly after biopsy the samples were frozen in liquid nitrogen, aliquoted in cryogenic vials and stored at −80 °C. Total liver lipids were determined by a gravimetrical method described by Starke et al. [19]. Briefly, total lipids were extracted for 24 h at 20 °C from homogenized tissue samples with *n*-hexane:isopropanol (mixing ratio 3:2) in different extraction steps followed by an evaporation step. The result was expressed in mg total lipid/g fresh liver weight.

#### 2.5.6. Activity and Behavior Monitoring

The fistulated cows (PG: *n* = 6, CG: *n* = 5) were alternately equipped with pedometers (total of four) and halters (total of five) including a noseband sensor (RumiWatch^®^, Liestal, Switzerland). RumiWatch^®^ pedometer is a 3D accelerometer recording the lying, standing, and walking times (WT). The noseband sensor consists of an oil-filled tube with a built-in pressure sensor, which responds to chewing movements, and thereby enables automatic measurements of the times of eating (ET) and ruminating (RT). The pedometers were alternately fastened to the right and left hind leg of the cows and the halters with the noseband sensor were fitted to each cow’s head individually. The cows had been familiarized with the technique before beginning the trial. After the recording period had been completed (3 ± 1 days per week), raw data were transferred via USB cable from the halter and pedometer to a personal computer using a specialized software (RumiWatch^®^ Manager 2, Version 2.1.0.0, ITIN + HOCH GmbH, Liestal, Switzerland). Raw data were then converted into 24 h-summaries using the novel converter software 0.7.3.11.

### 2.6. Calculations

To calculate the DMI of grass during full-time grazing the basic Equation (1) according to Heublein et al. [13] was used:

(1) $${\mathrm{DMI}}_{\mathrm{conc}4.5}(\mathrm{kg}/\mathrm{day})\;=\;({0.293\text{×}\mathrm{BW}}^{0.75}+3.14\text{×}\mathrm{ECM}-\mathrm{NEL}\;\mathrm{Conc}\text{×}4.5\;\mathrm{kg}\;\mathrm{Conc}\;\mathrm{DM})/\mathrm{NEL}\;\mathrm{pasture}\;$$

The calculation of feed intake during transition the intake of TMR was considered by modifying equation (1) accordingly. The concentrate term was replaced by the net energy for lactation (NEL) multiplied by the DMI of the ingredients of the TMR fed in confinement between week 2 to 5. Here, the TMR consisted of grass silage (GS), corn silage (CS), and concentrate (Conc TMR). Both equations. include the metabolic body size (kg BW^0.75^) and the energy-corrected milk yield (ECM).

(2) $${\mathrm{DMI}\;}_{\mathrm{week}\;2\;\mathrm{to}\;5\;}(\mathrm{kg}/\mathrm{day})\;=\;({0.293\text{×}\mathrm{BW}}^{0.75}+3.14\text{×}\mathrm{ECM}-(\mathrm{NEL}\;\mathrm{CS}\text{×}\mathrm{DMI}\;\mathrm{CS}\;+\mathrm{NEL}\;\mathrm{GS}\text{×}\mathrm{DMI}\;\mathrm{GS}+\mathrm{NEL}\;\mathrm{Conc}\;\mathrm{TMR}\text{×}\mathrm{DMI}\;\mathrm{Conc}\;\mathrm{TMR})/\mathrm{NEL}\;\mathrm{pasture}$$

Based on metabolic body weight (kg BW ^0.75^) and energy corrected milk yield (ECM) net energy requirement for maintenance was calculated according to the equations published by GfE [20], as described in detail by Drong et al. [6]. The data of BW, daily DMI, energy intake, energy balance (EB), milk yield, and milk composition were summarized to weekly means.

### 2.7. Statistical Analysis

Statistical analyses were performed using the Software SAS Enterprise Guide 7.1 (SAS Institute Inc., Cary, NC, USA). Variables recorded more than once a week were reduced to weekly means per cow before statistical analysis. For repeated measures, the MIXED procedure was used combined with a restricted maximum likelihood method. The model contained time (T) and group (G) as fixed factors as well as their interactions (GxT). Repeated measurements per animal were considered. Best fitting covariance structures and models were tested using the Akaike information criterion for a finite sample size. Effects were considered significant at *p* ≤ 0.05 while a trend was assumed for *p* ≤ 0.1. For each treatment, least squares means were calculated, and pairwise comparisons of each week were further evaluated, adjusted according to Tukey. For the variable serum triglyceride, which differed at the beginning of the trial, we used week 0 as covariate. Different letters indicate differences between weeks within particular experimental groups, whereas group differences in particular weeks were presented with different symbols. Results are presented as least square means and pooled standard error of means (PSEM) unless otherwise indicated (SD = standard deviation). Correlation coefficients between different traits were estimated using STATISTICA 13.0 (StatSoft Inc., Tulsa, OK, USA).

## 3. Results

### 3.1. Weather and Barn Climate Conditions

**Figure 1:**
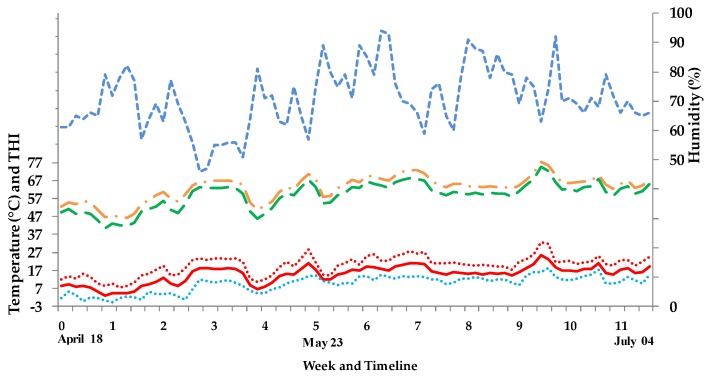
Daily outdoor average ambient temperature (solid line, red) with minima and maxima (dotted lines; blue, light red), outdoor humidity (small dashed line; blue), outdoor THI (dashed dot line, orange), and indoor THI (dash line; green). THI = temperature humidity indices; calculated according to Hahn [11]; THI = 0.8 td + RH×(td−14.4)+46.4, where td = dry bulb temperature (°C) and RH = relative humidity.

Outdoor daily average ambient temperature with minima and maxima, outdoor humidity, and outdoor and indoor THI are illustrated in Figure 1. 

The THI was generally 4.0 ± 0.6 (mean ± SD) units higher indoors compared to outdoors, whereby the average daily THI was 58.8 ± 6.1 outdoors and 62.8 ± 5.6 indoors. Periods of mild heat stress [21,22,23] were detected outdoors in week 6 and 9 (THI between 65 and 70) and indoors in week 6, 7, and 9–11 (THI between 65 and 75).

### 3.2. Feed Composition

The ingredients and chemical diet composition are documented in Table 1 and 2.

The DM of grass was lower compared to TMR fed in confinement. The CP value of grass varied but was higher compared to the TMR. For NEL and sugar concentration a time-dependent decrease was noticed. From week 4 onwards the NEL concentration of pasture was lower compared to TMR. During full-time grazing the amount of sugar was on average 5 times higher compared to TMR. During full-time grazing the CF concentration was on average 36% higher compared to TMR.

### 3.3. Paddock Characteristics

All four paddocks were on average covered to 79.5 ± 13.6% by grass, 7.5 ± 6.9% by forbs, and 4.4 ± 7.4% by legumes (estimated pasture coverage; mean ± SD). In Table A1, each paddock and the recorded plant species and proportions are characterized. The average pasture height upon entering the pasture (pregrazing) was 14.3 ± 2.6 cm (mean ± SD) and 7.9 ± 1.1 cm when rotating to the next paddock (postgrazing). The average pasture allowance amounted to 142 ± 46 kg of DM grass/cow/day in the beginning of each grazing period and decreased to 87 ± 18 kg DM grass/cow/day after 6 days of grazing in mean.

### 3.4. Animal Measurements

DMI average feed and water intake variables are presented in Table 3. The average DMI of the CG was 22.0 ± 0.5 kg DM/cow/day. The average water intake of the CG was 85.5 ± 2.8 kg/cow/day. During week 2 to 5 TMR intake of the PG declined from 20.6 to 11.0 ± 0.2 kg of DM/cow/day. The same applied for the water intake from week 2 to 5. During transition and on a full grazing ration the DMI of pasture was lower compared to TMR intake of CG. The calculation according to Heublein et al. [13] and the n-alkane method showed comparable DMI results, whereas the calculation by the exclosure cage method was characterized by high variation.

### 3.5. Milk Yield and Composition

**Figure 2:**
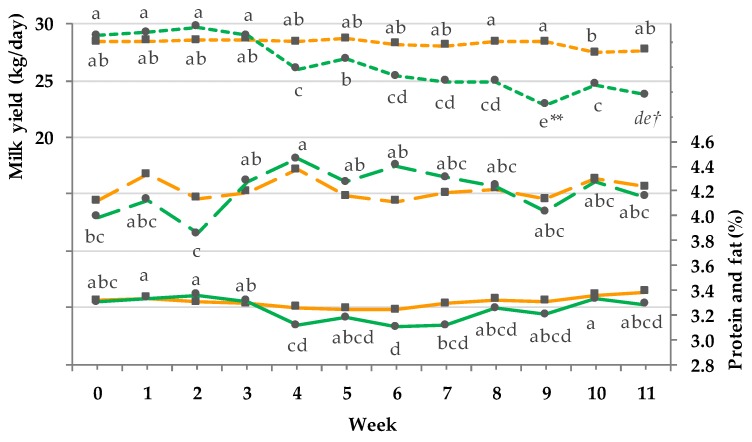
Effect of a ration change from an indoor-based total mixed ration (TMR) to pasture on milk yield (kg/day, short dashed line, pooled SEM = 0.9), fat (%, long dashed line; pooled SEM = 0.1), and protein (% solid line; pooled SEM = 0.04); ■ = confinement group (CG, *n* = 31; orange); ● = pasture group (PG, *n* = 26; green). Significance: milk yield kg/day= group (G): P = 0.104, time (T): *p* < 0.001, GxT: *p* < 0.001; fat% = G: *p* = 0.943, T: *p* < 0.001, GxT: *p* < 0.001; protein% = G: *p* <0.001, T: *p* < 0.001, GxT: *p* < 0.05. Different symbols indicate significant differences between groups in particular week (** *p* ≤ 0.01; † *p* ≤ 0.1); different letters (a–e) indicate significant differences between weeks within particular groups (*p* ≤ 0.05). The CG stayed on a TMR-based diet during the entire trial, while the PG was slowly introduced to a pasture-based ration: week 0 and 1 = TMR, week 2 = TMR and 3 h pasture/day, week 3 and 4 = TMR and 12 h pasture/day, and week 5 to 11 = pasture and 4.5 kg DM concentrate/cow/day.

In Figure 2 the variables milk yield, protein, and fat yield are illustrated. 

The milk yield of the PG significantly decreased commencing with week 4 (12 h pasture plus TMR ad libitum) until the end of the trial, while milk yield of CG remained on a stable level (GxT: *p* < 0.001). For the variable protein (%) G and T effects and a GxT interaction were documented (milk protein (%) = *p*_G_ < 0.001, *p*_T_ < 0.001, *p*_GxT_ < 0.05) because of a numerically decrease, incipient in week 4. The variable fat (%) was influenced by a T and GxT interaction (milk fat (%) = *p*_G_ = 0.943, *p*_T_ < 0.001, *p*_GxT_ < 0.001), showing a numerical increase between week 3 and 8. Milk lactose content exhibited a T effect and a GxT interaction without showing a single G effect (milk lactose content = *p*_G_ = 0.361, *p*_T_ < 0.001, *p*_GxT_ < 0.001; data not shown). In the PG lactose content continuously decreased beginning with week 3 (4.8 ± 0.03; LSmean ± PSEM), whereas the lactose content of the CG fluctuated around the starting value (4.9 ± 0.03; LSmean ± PSEM). No significant differences were observed between the groups at corresponding time points.

### 3.6. BW and BCS.

**Figure 3:**
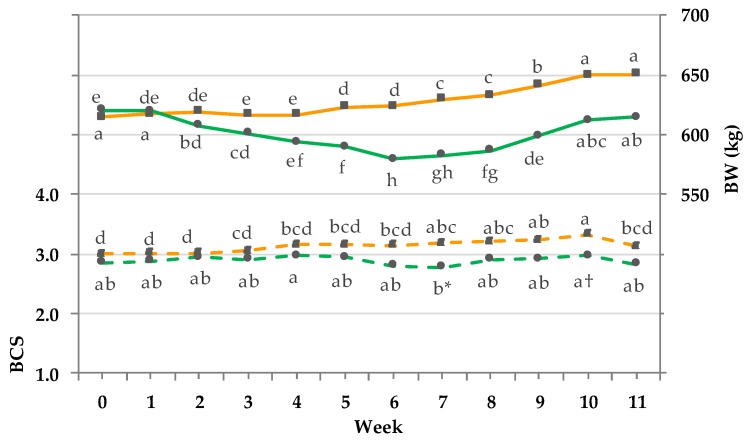
Effect of a ration change from an indoor-based total mixed ration (TMR) to pasture on body weight (BW) kg (solid line, pooled SEM = 11) and body condition score (BCS) (dashed line; pooled SEM = 0.07); ■ = confinement group (CG, *n* = 31; orange); ● = pasture group (PG, *n* = 26; green). Significance: BW = group (G): *p* < 0.1, time (T): *p* < 0.001, GxT: *p* < 0.001; BCS = G: *p* < 0.05, T: *p* < 0.001, GxT: *p* < 0.001. Different letters (a–h) indicate significant differences between weeks within particular groups (*p* ≤ 0.05). Because of technical problems in week 9 the average BW of week 8 and 10 was assumed for both groups. The CG stayed on a TMR-based diet during the entire trial, while the PG was slowly introduced to a pasture-based ration: week 0 and 1 = TMR, week 2 = TMR and 3 h pasture/day, week 3 and 4 = TMR and 12 h pasture/day, and week 5 to 11 = pasture and 4.5 kg DM concentrate/cow/day.

In the first two weeks of the trial, both groups started with similar BW (Figure 3).

The BW of the CG showed from week 4 on until the end of the trial a steady increase by 34 ± 10 kg (means ± SD). With the beginning of pasture access in week 2 (3 h pasturing/day) the BW of the PG showed a decrease until week 6 (full-time pasturing) by 40 ± 11 kg. Thereafter, BW increased again until the end of the trial (35 ± 11 kg). The BCS of the PG slightly increased by 0.1 ± 0.07 units until week 4 (12 h pasturing/day). Then, until week 7 (full-time pasturing) a decrease by 0.2 ± 0.07 units was observed, accompanied by a significant difference between groups (*p* <0.01). In the second half of the trial, an increase up to the initial value of the beginning of the trial occurred (PG average increase in BCS units: 0.2 ± 0.07, week 7 to 11).

### 3.7. Hematology 

Red blood cell (RBC) count showed a GxT interaction (Table 4). The RBC concentration of the PG significantly increased with the beginning of week 3, reaching the highest value in week 6, followed by a decrease. In week 10, a tendency for a different RBC was observed between the groups. Nearly the same development was observed for the concentration of hemoglobin (HGB) and hematocrit (HCT). Both variables showed a GxT interaction (HGB = *p*_GxT_ < 0.001; HCT = *p*_GxT_ < 0.001). The concentration of HGB of the PG showed a continuous increase reaching a peak value in week 11, which significantly differed to the concentration of the CG. In the PG, the HCT in week 0 significantly differed from week 6 because of a time-dependent increase, followed by a decrease not reaching the initial value. The CG showed a continuous and slight decrease in HCT over the course of the trial. For the variables of mean corpuscular hemoglobin (MCH) and mean corpuscular hemoglobin concentration (MCHC) a significant GxT interaction was observed (MCH = *p*_GxT_ < 0.001; MCHC = *p*_GxT_ < 0.001). The PG showed a continuous and more pronounced increase, reaching the highest concentration in week 11, which significantly differed to week 0. The MCH and MCHC concentrations of the PG peaked in week 5 and decreased afterwards. The mean corpuscular volume (MCV) of the PG peaked in week 5 and then decreased again without reaching the initial values. The MCV of the CG did not change until week 7, then, showed a numerical increase until the end of the trial, resulting in a tendency of GxT interaction (MCV = *p*_GxT_ < 0.1). For the parameter red blood cell distribution width (RDW) a tendency was observed for the GxT interaction. Both groups exhibited an increase, the PG until week 5, the CG until week 11. Between week 6 and 11 (pasture plus concentrate), no significant difference within the PG was observed. Platelet distribution width (PDW) increased in both groups resulting in a GxT interaction (PDW = *p*_GxT_ < 0.01). The concentration of both groups increased continuously but time dependently until the end of the trial.

### 3.8. Clinical-Chemical Traits

**Figure 4:**
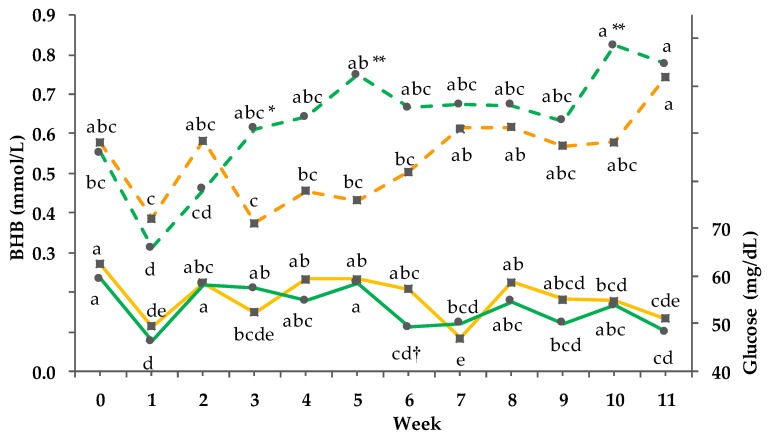
Effect of a ration change from an indoor-based total mixed ration (TMR) to pasture on serum glucose (solid line, pooled SEM = 1.6) and beta-hydroxy-butyrate (BHB) (dashed line; pooled SEM = 0.04); ■ = confinement group (CG, *n* = 21; orange); ● = pasture group (PG, *n* = 22; green). Significance levels: glucose = group (G): *p* < 0.05, time (T): *p* < 0.001, GxT: *p* < 0.01; BHB = G: *p* < 0.001, T: *p* < 0.001, GxT: *p* < 0.001. Different symbols indicate significant differences between groups in particular week (** *p* ≤0.01; * *p* ≤ 0.05); different letters (a–e) indicate significant differences between weeks within particular groups (*p* ≤ 0.05). The CG stayed on a TMR-based diet during the entire trial, while the PG was slowly introduced to a pasture-based ration: week 0 and 1 = TMR, week 2 = TMR and 3 h pasture/day, week 3 and 4 = TMR and 12 h pasture/day, and week 5 to 11 = pasture and 4.5 kg DM concentrate cow/day.

**Figure 5:**
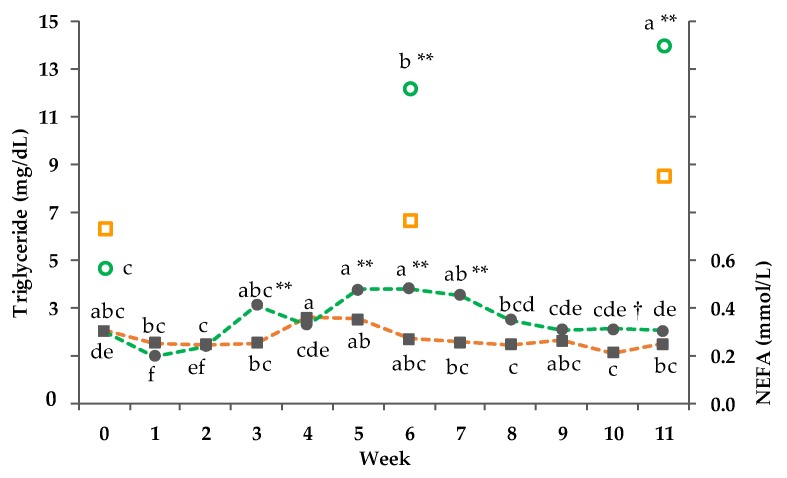
Effect of a ration change from an indoor-based total mixed ration (TMR) to pasture on serum triglyceride (no line, pooled SEM = 11) and non-esterified fatty acids (NEFA) (dashed line; pooled SEM = 0.02); ■□ = confinement group (CG, *n* = 21; orange); ●○ = pasture group (PG, *n* = 22, green). Significance levels: triglyceride = group (G): *p* < 0.01, time (T): *p* < 0.001, GxT: *p* < 0.001, Week 0 was set as covariate; NEFA = G: *p* < 0.001, T: *p* < 0.001, GxT: *p* < 0.001. Different symbols indicate significant differences between groups in particular week (** *p* ≤0.01; † *p* ≤ 0.1); different letters (a–f) indicate significant differences between weeks within particular groups (*p* ≤ 0.05). The CG stayed on a TMR-based diet during the entire trial, while the PG was slowly introduced to a pasture-based ration: week 0 and 1 = TMR, week 2 = TMR and 3 h pasture/day, week 3 and 4 = TMR and 12 h pasture/day, and week 5 to 11 = pasture and 4.5 kg of DM concentrate/cow/day

**Figure 6:**
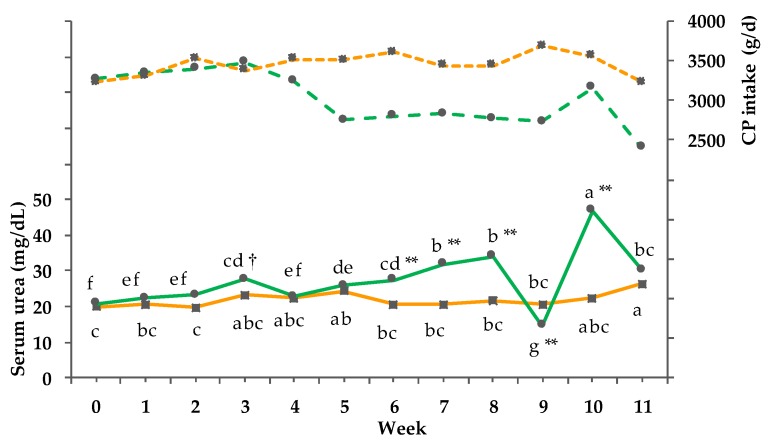
Effect of a ration change from an indoor-based total mixed ration (TMR) to pasture on serum urea concentrations (solid line, pooled SEM = 0.90) and protein (CP) intake (dashed line); ■ = confinement group (CG, *n* = 21; orange); ● = pasture group (PG, *n* = 22; green). Significance levels: serum urea = group (G): *p* < 0.001, time (T): *p* < 0.001, GxT: *p* < 0.001. Different symbols indicate significant differences between groups in particular weeks (** *p* ≤ 0.01); different letters (a–g) indicate significant differences between weeks within particular groups (*p* ≤ 0.05). The CG stayed on a TMR-based diet during the entire trial, while the PG was slowly introduced to a pasture-based ration: week 0 and 1 = TMR, week 2 = TMR and 3 h pasture/day, week 3 and 4 = TMR and 12 h pasture/day, and week 5 to 11 = pasture and 4.5 kg DM concentrate cow/day.

Data of the clinical chemistry are documented in Table 5 and Figure 4, Figure 5 and Figure 6. The serum variables albumin, AST, γ-GT, and GLDH were not influenced by treatments. Serum cholesterol and serum total protein showed only a time dependency. Serum bilirubin of the PG increased more pronouncedly from week 0 to 6 compared to the CG and was followed by a decrease from week 6 to 11, which explains the GxT interaction. Within the PG no significant difference between week 0 and 11 but between week 0 and 6 as well as week 6 and 11 was observed. 

A significant GxT interaction was detected for the concentration of serum BHB (*p*_GxT_ < 0.001). In week 3, 5, and 10 the BHB concentration of the PG was significantly higher compared to the concentration of the CG. In week 11, the BHB concentration significantly differed from the initial values within the PG (*p* < 0.01). For glucose, a significant interaction between GxT was observed (*p*_GxT_ < 0.001). With the exception of week 3 and 7, the glucose concentration of the PG was lower compared to the concentration of the CG. The lowest glucose level within the PG was measured three times during the trial (week 6, 9, 11; 49.2 ± 1.6 mg/dL (mean ± SD)). There was no significant correlation between glucose and BHB (r = 0.09; *p* = 0.779). However, a significant correlation of BHB with THI was detected (r = 0.82; *p* = 0.001). 

Furthermore, between week 2 to 6 we observed an increase of the NEFA concentration of the PG from 0.24 to 4.48 ± 0.02 mmol/L (Figure 5). In week 3, 5, 6, and 7 the concentration of NEFA significantly differed from the CG, showing the highest values in week 5 and 6, followed by a decrease before reaching the starting level. Serum triglycerides increased time-dependently in both groups, whereby the increase was more pronounced in the PG, causing a significant GxT interaction. In week 11, the triglyceride concentration of the PG was three times higher in comparison to week 0. Within the PG, week 0 differed significantly from week 6 and 11 (*p* < 0.001). Moreover, significant differences were found in all weeks between the groups. 

A continuous increase of serum urea concentrations in the PG was observed up to a temporary decline in week 9 and 11 (Figure 6). The increase of the CG was less pronounced, resulting in a significant GxT interaction. Within the PG, significant differences were observed from week 6 to 10. Serum urea concentrations did not correlate significantly with pasture CP content (r = 0.32; *p* = 0.326), but with milk urea content (r = 0.56; *p* <0.001). 

### 3.9. Net energy Intake and Balance

**Figure 7:**
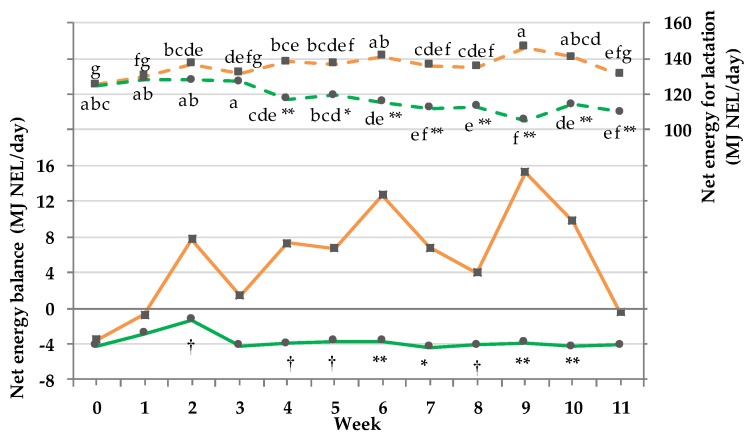
Effect of a ration change from an indoor-based total mixed ration (TMR) to pasture on net energy balance (NEB: MJ NEL/day) and net energy of lactation intake (NEL: MJ/day). Dry matter intake (DMI) on pasture based on the calculation according to equations (1) and (2). Solid line = NEB, pooled SEM = 2.1 and short dashed line = NEL, pooled SEM = 2.6; ■ = confinement group (CG, *n* = 31; orange); ● = pasture group (PG, *n* = 26; green). Significance levels: NEB = group (G): *p* < 0.001, time (T): *p* <0.001, GxT: *p* <0.001; NEL = G: *p* <0.001, T: *p* <0.001, GxT: *p* <0.001. Different symbols indicate significant differences between groups in a particular week (** *p* ≤ 0.01; * *p* ≤ 0.05; † *p* ≤ 0.1); different letters (a–f) indicate significant differences between weeks within particular groups (*p* ≤ 0.05). The CG stayed on a TMR-based diet during the entire trial, while the PG was slowly introduced to a pasture-based ration: week 0 and 1 = TMR, week 2 = TMR and 3 h pasture/day, week 3 and 4 = TMR and 12 h pasture/day, and week 5 to 11 = pasture and 4.5 kg DM concentrate cow/day.

A positive EB was more pronounced in the CG as shown in Figure 7. The EB of the PG never reached a positive value during the whole trial, resulting in a GxT interaction (*p*_GxT_ < 0.001). The NEL intake of the PG decreased significantly within the group at the beginning of week 4 and did not reach its starting value again. The NEL intake of the CG fluctuated only minimally throughout the trial, resulting in a GxT interaction (*p*_GxT_ < 0.001). Significant differences between both groups were observed from week 4 until the end. The lowest NEL was documented in the PG at week 9, whereas the CG showed the highest NEL (106 vs. 146 ± 2.6 MJ NEL/day).

### 3.10. Fat Depots and Liver Fat

**Figure 8:**
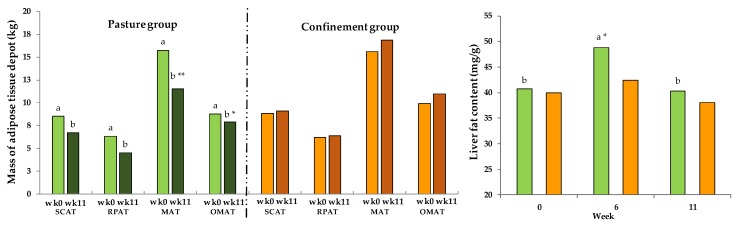
Effect of a ration change from an indoor-based total mixed ration (TMR) to pasture on body fat depots, in week 0 and 11 (left diagram; pooled SEM subcutaneous adipose tissue (SCAT) = 0.73; SEM omental adipose tissue (OMAT) = 0.63; SEM retroperitoneal adipose tissue (RPAT) = 0.56; SEM mesenteric adipose tissue (MAT) = 0.88) and liver fat content (right diagram; pooled SEM = 1.7); confinement group (CG, *n* = 16; orange); pasture group (PG, *n* = 16; green). SCAT = subcutaneous adipose tissue, RPAT = retroperitoneal adipose tissue, MAT= mesenteric adipose tissue, OMAT = omental adipose tissue. Significance: SCAT = group (G): *p* < 0.186, time (T): *p* < 0.05, GxT: *p* < 0.01; RPAT = G: *p* < 0.209, T: *p* < 0.05, GxT: *p* < 0.01; MAT = G: *p* < 0.05, T: *p* < 0.1, GxT: *p* < 0.01; OMAT = G: *p* < 0.05, T: *p* < 0.05, GxT: *p* < 0.01; liver fat content = G: *p* < 0.1, T: *p* < 0.001, GxT: *p* < 0.05. Different symbols indicate significant differences between groups in a particular week (** *p* ≤ 0.01; * *p* ≤ 0.05); different letters (a,b) indicate significant differences between weeks within particular groups (*p* ≤ 0.05). The CG stayed on a TMR-based diet during the entire trial, while the PG was slowly introduced to a pasture-based ration: week 0 and 1 = TMR, week 2 = TMR and 3 h pasture/day, week 3 and 4 = TMR and 12 h pasture/day, and week 5 to 11 = pasture and 4.5 kg DM concentrate cow/day.

Calculated masses of fat depots per group and time are presented in Figure 8. The SCAT of the PG decreased over the entire experimental period by 1.8 kg. The same picture shows the RPAT. The maximum body tissue loss occurred for the MAT of the PG with 4.3 kg. The lowest decrease of the PG was shown for OMAT with 0.9 kg. In total, the PG lost 8.7 kg of body fat, calculated from the sum of the measured depots. The increase in body fat depots was more pronounced in the CG gaining 2.9 kg of body fat in total. Significant differences between both groups were observed in week 11 for the fat depots MAT and OAT, as well as a RPAT. Overall, for fat depots, a GxT interaction was observed (SCAT = *p*_GxT_ < 0.05; RPAT = *p*_GxT_ < 0.05; MAT = *p*_GxT_ < 0.05; OMAT = *p*_GxT_ < 0.05). The results of the total lipid analysis in liver tissue are shown in Figure 8. 

The total liver fat content of the PG increased from week 1 to 6 and decreased afterwards. The CG showed the same development, but less pronounced, resulting in GxT interaction (*p*_GxT_ < 0.05). A significant difference between both groups was observed after introduction to full-time grazing in week 6 (*p* < 0.05).

### 3.11. Activity and Behavior Monitoring 

**Figure 9:**
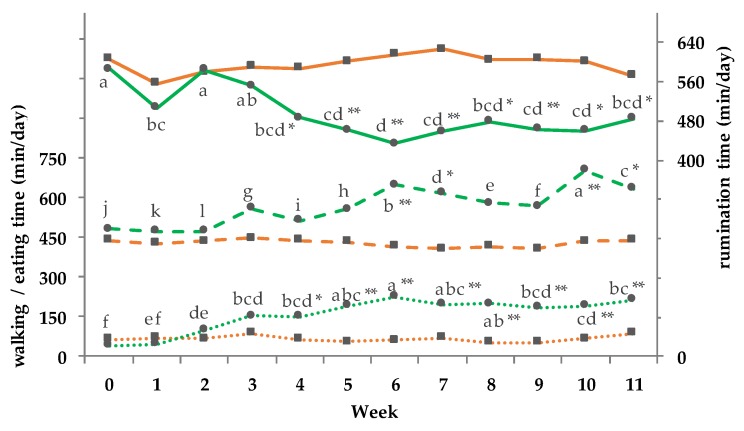
Effect of ration from an indoor-based total mixed ration (TMR)to pasture on walking (pointed line; pooled SEM = 17), eating (dashed line; pooled SEM = 34), and rumination (solid line; pooled SEM = 30) time min/day; ■ = confinement group (CG, *n* = 5; orange); ● = pasture group (PG, *n* = 6; green). Significance levels: Walking time = group (G): *p* < 0.01, time (T): *p* < 0.05, GxT: *p* < 0.05; eating time = group (G): *p* < 0.001, time (T): *p* < 0.01, GxT: *p* < 0.01; rumination time = group (G): *p* < 0.001, time (T): *p* < 0.210, GxT: *p* < 0.05. Different symbols indicate significant differences between groups in a particular week (** *p* ≤ 0.01; * *p* ≤ 0.05); different letters (a–l) indicate significant differences between weeks within particular groups (*p* ≤ 0.05). The CG stayed on a TMR-based diet during the entire trial, while the PG was slowly introduced to a pasture-based ration: week 0 and 1 = TMR, week 2 = TMR and 3 h pasture/day, week 3 and 4 = TMR and 12 h pasture/day, and week 5 to 11 = pasture and 4.5 kg DM concentrate cow/day.

WT, ET, and RT are illustrated in Figure 9. For WT, ET, and RT a group and a GxT interaction was found, a T effect was documented only for WT and ET (WT = *p*_G_ < 0.01, *p*_T_ < 0.05, *p*_GxT_ < 0.05; ET = *p*_G_ < 0.01, *p*_T_ < 0.01, *p*_GxT_ < 0.01; RT = *p*_G_ < 0.01, *p*_T_ = 0.210, *p*_GxT_ < 0.05). With the beginning of pasture access in week 2 the WT increased continuously in the PG until week 6 and decreased slightly again thereafter. From week 4 to 11 a significantly higher WT compared to the CG was observed. From week 3 onwards the PG spent on average 569 ± 18 min/day eating, compared to the CG with 429 ± 20 min/day. The RT decreased in the PG from week 2 until week 6 and exhibited a slight increase until the end of the trial. Significant differences between the groups were present during full-time grazing from week 4 to 11. A significantly positive correlation was observed for WT and ET in the PG from week 2 until 11 (r = 0.80; *p* = 0.01), as well as between WT and blood HCT from week 0 to 11 (r = 0.86; *p* = 0.001).

## 4. Discussion

The aim of the present experiment was to test the efficacy of supplementing high-yielding Holstein cows raised on a rotational all-day pasture system with moderate amounts of concentrate feed (4.5 kg DM cow/day) as an instrument to compensate for the energy shortage usually observed during transition [5,13].

### 4.1. Effects of Transition to Grazing on DMI

The average DMI (22 kg/day) and milk production (28 kg/day) of the CG showed the usual performance of the cows during mid-lactation when an appropriate balanced ration is fed ad libitum. To estimate the DMI for the PG we used different methods in order to improve the calculations based on DMI, like energy balance. The calculation method of Heublein et al. [13] nearly matched the DMI estimation of the n-alkane method (week 6 and 7), while the exclosure cage calculations revealed partly nonreliable DMI estimations. This agrees with earlier findings by Schären et al. [5] and Undi et al. [24]. Therefore, the results of the exclosure cages need to be interpreted carefully. With increasing time on pasture, the DMI of grass increased, stayed more or less constant during full-time pasturing and was 3 kg lower (pasture + concentrate feed) compared to CG. The capacity for DMI from pasture depends on several factors (e.g., pasture availability and pasture height). Compared to a continuous grazing system [5] the rotational grazing system, with an increased average pasture height, daily pasture allowance, and frequency of pasture allocation as applied in the present study, did not increase the DMI of PG. However, it needs to be considered that the higher concentrate feed allowance, as practiced in the present experiment, might partly have displaced pasture intake. Moreover, the chemical composition of the pasture influences feed intake as well. Decruyenaere et al. [25], Roca Fernandez, and Gonzalez Rodriguez [26] discussed low DMI from pasture in the context with chemical composition of the feed. They concluded that a pasture DM content of ≤ 20% resulted in a higher rumen fill with water, which had a negative impact on the DMI. In the present trial, the pasture DM was less than 20% with exception of week 3. Furthermore, with decreasing pasture quality, the organic matter digestibility (OMD) usually begins to decrease whereas the rumen content increases due to slower digestion.

### 4.2. Changes of Grazing Behavior and Its Effect on DMI

The decrease in DMI of PG also coincided with an increase of time spent for eating (ET: plus 138 min/day), which suggests that the cows probably spent more time during feed acquisition from pasture compared to the CG. The increase in ET can be explained by a limitation of feed quantity per bite [27]. Parker [28] explained that the average biting rate of grazing cows is 60 bites/min and that the bite size varies from 0.62 to 0.19 g DM/bite. In our trial, the PG spent on average 617 min/day eating on average (week 5 to 11, pasture plus concentrate feed). Assuming an average bite size of 0.41 g/DM bite and 60 bites/min based on Parker [28] our results would mean that the cows had eaten approximately 15 kg/day DM grass, which fits to the calculated average DMI of 13 kg of grass (week 5 to 11) according to equations (1) and (2). 

### 4.3. Effects of DMI on Body Condition

Constant changes in nutrients due to weather influences along with a low DMI resulted in a decrease in NEL intake of 11% (relative deviation) during full-time grazing compared to week 0 and 1 (TMR only). This was paralleled by a decrease in BW and BCS from week 1 to 6. A major part of BW decreasing was probably due to a lower rumen content when cows turned to pasture which agrees with earlier findings [5]. On the other hand, the lower degree of rumen fill would rather hint at grazing time as a DMI limiting factor. Moreover, rumen fill might only explain a part of the BW loss. Decrease of BCS during transition and of body fat depots support this view and suggest an energy deficit.

### 4.4. Effect of Behavioral Adaption on Energy Metabolism

In our trial changes in behavior and metabolism during adaption to pasture were clearly visible in the different monitored variables. For example, the rumination time decreased while the physical activity was intensified over the weeks. For dry cows less RT could also be documented by Schirmann et al. [29] who argued that rumination and lying times are positively associated. Our trial lead to the assumption that more ET combined with more activity, as well as a lower DMI and, thus, rumen content, were the main factors for reduced RT.

Kaufmann et al. [30] observed a higher energy expenditure of cows on pasture, positively correlated with walking and eating time. Grazing and walking increased the energy maintenance requirement by 1 to 50% [20,31]. Intensified activity, together with the lower energy intake resulted in a constant moderate NEB for the PG (week 5 to 11: −4.1 MJ NEL/day) compared to the average value of CG (5.52 MJ NEL/day). Thus, the concentrate supply of 4.5 kg DM/cow/day during full-time grazing failed to fully compensate the energy shortage. Moreover, in addition to behavioral adaption, the cows’ rumen microbiota has to adjust to the new feed [32] with subsequent effects on nutrient utilization [33]. Schären et al. [32] observed the influence of the aforementioned adaption concerning rumen microbiota indicating a decreased fermentation activity and thereby causing an energy deficit. 

Schären et al. [5] further documented metabolic adaptions concerning low blood glucose levels during high temperatures. They assumed that cows avoid metabolic heat production by blocking adipose tissue mobilization and increasing glucose burning as energy source. Despite temperature influences (week 9), blood glucose concentration showed (in our trial) no significant differences compared to the CG indicating that blood glucose homeostasis was not disturbed in the present experiment. A significant increase in NEFA of the PG indicates that lipomobilization occurred. The cows obviously mobilized body reserves to meet their nutritional requirements, which was also reflected by changes of body fat depots along with the increases in NEFA and BHB concentrations as well as of the liver fat content. Thus, we could demonstrate that NEB in mid-lactating cows is also associated with fat depot changes that are comparable to early lactation [7,34]. The highest loss of fat depot showed MAT. MAT, being a visceral fat depot, has been shown in human studies to exhibit different metabolic characteristics compared to the other fat depots [35]. In total, the PG lost approximately 8.7 kg of body fat. Taking into account the energy concentration of fat of 39.8 kJ/g [36] and assuming that 84% of the mobilized energy was converted into net energy for milk synthesis [37], the PG used 3.5 MJ body fat/day for milk energy, which is close to the calculated EB during the trial period of week 5 to 11. We also observed an increase in liver fat content of the PG at week 6. This coincides with a significant increase of total bilirubin in serum, possibly hinting at a compromised liver function around week 6 of experiment. However, in light of the fact that the other biochemical traits associated with liver function and hepatocyte integrity such as albumin, cholesterol, AST, GGT, and GLDH remained unaltered, the temporal increase in liver fat and total bilirubin can be interpreted as adaptation rather than a liver damage, confirming the results of Schären et al. [5]. Besides the effect of intensified physical activity on energy maintenance requirement, the RBC count clearly reflected metabolic adaptations as an increase in RBC, HCT, and HGB as well as MCV was noticed, which suggests an improved oxygen supply of the PG (r = 0.84; *p* < 0.001 for WT:HCT). Another reason for the increased HCT could be a possible decrease in water intake, which, however, could not be measured in the present experiment. 

### 4.5. Effect of Transition on Milking Performance

The loss of milk production started in the second week of transition, confirming an insufficient nutrient intake. The effect of a rotational grazing system only resulted in a short-term increase in milk yield with every paddock rotation (data not shown). Abrahamse et al. [38] documented that strip grazing improves productivity in cows. Other studies showed that a daily rotational grazing system results in a higher milk production because of a greater DMI when the pasture allowance is large. When herbage allowance is high, the milk production reaches a plateau at 4 kg concentrate feed supplementation; but with restricted pasture allowance, a linear response up to 6 kg of concentrate was found by Delaby et al. [39]. Independent of weather and chemical grass composition, concentrate supplementation during full-time pasturing did not lead to the same milk yield as in the CG.

The observed milk fat increase is contrary to other observations on pasture [40,41] but could be an effect of increased fiber intake, underlining the effects of different grazing systems [5].

The significant increase in serum and milk urea concentration during full-time pasturing compared to confinement housing could not be explained by increased CP intake (CP: 2778 vs. 3312 ± 326 g/day (means ± SD)). In contrast, Bargo et al. [42], observed a positive association between serum urea levels and pasture protein content. Despite higher CP concentrations of pasture, Santana et al. [43] and Kohn [44] reviewed that milk or blood urea N are indicators for diet adequacy and nitrogen utilization efficiency in lactating dairy cattle. This underlines the energy deficit of the PG and rumen microflora of PG cows, resulting in ammonia excessively passing into the bloodstream and being converted to urea in the liver, or leading to an increased endogenous amino acids catabolism. The drop in blood serum and milk urea found at the beginning of week 9 in our trial could possibly be consequence of a sudden pronounced change in pasture components (lower RNB and CP content) from week 8 to 9. However, another reason could be an increased THI. Wilson et al. [45] assumed that the OMD of grass correlates negatively with temperature during regrowth of grass, which would contribute to an energy deficiency of the microorganisms because of a decreasing passage rate and as a consequence to a reduced DMI. The calculated DMI of week 9 according to equations (1) and (2) support this assumption.

## 5. Conclusions

Transition from confinement to pasture plus moderate concentrate supplementation of 4.5 kg DM/day showed complex nutritional and metabolic adaptions. Despite a moderate concentrate feed supply and weekly paddock rotation it was not possible to counterbalance the lower energy intake from pasture for the first 6 weeks on a full grazing ration. Compared to the CG the PG consumed less DM during and after transition, resulting in a nutrient deficiency. The cows responded to this situation with lower milking performance, mobilizing body fat depots, and NEB, accompanied by substantial changes in animal behavior. The PG spent more time for eating and walking, resulting in less time for ruminating. The higher physical activity of the PG on pasture compared to CG was reflected in different variables of the red blood cell count as well as a habituation effect. 

Further research is needed to investigate whether a more frequent rotation in relation to timing of grazing would attenuate the energy deficiency, or if the transition can be managed more gradually, supplying grass already during confinement before full-time pasturing starts.

## Figures and Tables

**Table 1:** Ingredients of concentrate.

Ingredient (% DM)	Concentrate TMR CG	Concentrate TMR PG	Concentrate PG
Soyabean meal	20.7	20.7	-
Rapeseed meal	14.0	14.0	-
Wheat	18.2	18.1	29.6
Corn	18.2	18.1	29.6
Barley	18.2	18.1	29.6
Dried sugar beet pulp	5.0	5.0	-
Feed lime	1.9	1.9	-
Soyabean oil	1.0	1.0	1.0
MgO	-	0.3	1.2
Urea	1.0	1.0	-
NaCl	0.3	0.3	-
Mineral feed ^1^	1.5	1.5	9.0

DM = dry matter, TMR = total mixed ration, CG = confinement group, PG = pasture group. ^1^ Per kilogram mineral feed: 140 g Ca; 120 g Na; 70 g P; 40 g Mg; 6 g Zn; 5.4 g Mn; 1 g Cu; 100 mg I; 40 mg Se; 25 mg Co; 1,000,000 IU vitamin A; 100,000 IU vitamin D3; and 1500 mg vitamin E.

**Table 2:** Chemical composition of experimental diets.

Item (g/kg of DM)	TMR	TMR	Concentrate	Pasture (per week)									
	CG	PG	PG										
	(Week 0 to 11)	(Week 0 to 4)	(Week 5 to 11)	2	3	4	5	6	7	8	9	10	11
DM (g/kg)	465	477	899	196	217	198	164	147	183	145	147	167	172
Ash	66	6s6	108	80	82	85	90	105	90	105	102	93	105
Cp	157	159	93	213	199	188	178	168	201	168	198	209	155
UCp	152	151	148	154	153	142	143	131	150	126	142	143	133
NEL (MJ/kg of DM) *	6.8	6.8	7.6	6.9	6.8	6.4	6.3	5.7	6.5	5.6	6.1	6.1	5.9
Sugar	18	18	27	222	170	128	92	79	97	102	87	83	83
Starch	250	255	579	-	-	-	-	-	-	-	-	-	-
RNB	0.03	−0.1	−8.9	6.0	6.9	4.1	6.0	6.0	9.1	2.3	9.0	8.8	4.2
CF	184	184	31.5	157	189	211	254	262	238	249	242	236	271
NDF_OM_	363	363	124	377	433	472	509	514	462	479	472	468	521
ADF_OM_	206	203	42	174	208	233	278	288	255	277	270	261	300
EE	37	36	30	49	46	38	34	36	42	43	45	48	31

TMR = total mixed ration, CG = confinement group; PG = pasture group; DM = dry matter, CP = crude protein, uCP = utilizable crude protein; NEL* = net energy lactation (MJ/kg DM), RNB = ruminal nitrogen balance; CF = crude fiber; NDF_OM_ = neutral detergent fiber; ADF_OM_ = acid detergent fiber; EE = ether extract. NDF and ADF were expressed without residual ash and are therefore referred to as NDF_OM_ and ADF_OM_.

**Table 3:** Grazing management conditions and sward intake per cow calculated by means of the n-alkane method and the exclosure cages, as well as according to Equations (1) and (2).

Week		0	1	2	3	4	5	6	7	8	9	10	11	PSEM ^3^	*p*-Value		
Week of Paddock Rotation *					3–4		5–6.5		6.5–7.5	7.5–8.5	8.5–9.5	9.5–10.5	10.5–11		Group (G)	Time (T)	GxT
CG/kg/day)	Water intake	83	91	85	86	82	86	88	82	89	87	86	81	2.4	<0.001	<0.001	<0.001
PG (kg/day)		80	76	67	46	31	-	-	-	-	-	-					
CG (kg/day DMI)	TMR	20.5	21.0	22.5	21.5	22.4	22.4	23.0	21.9	21.9	23.5	22.7	20.6	0.5	<0.001	<0.001	<0.001
PG (kg/day DMI)	TMR	20.6	21.1	17.6	13.0	11.0											
	PI ^1^ Alkane							14.6	12.8								
PI ^1^ Alkane + concentrate								19.1	17.3								
PI ^1^ according to equation (1) + (2)				2.8	7.1	7.9	13.1	14.2	12.0	14.0	11.7	13.1	12.8	0.4	<0.001	<0.001	<0.001
PI ^1^ Heublein + TMR + concentrate ^2^				20.4	20.1	18.9	17.6	18.7	16.5	18.5	16.2	17.6	17.3	0.4	<0.001	<0.001	<0.001
PI^1^ exclosure cage *					15.8 ± 3.4		21.9 ± 2.4		15.3 ± 1.8	19.4 ± 7.4	18.5 ± 1.7	25.6 ± 7.5	20.0 ± 4.9				
PI ^1^ exclosure cage + concentrate ^2,^*							26.4 ± 2.4		19.8 ± 1.8	20.9 ± 7.4	22.9 ± 1.7	30.1 ± 7.5	24.5 ± 4.9				

^1^ Pasture intake. ² Four-and-a-half kilograms (DM) concentrate cow/day; divided into two portions from week 5 on. ^3^ PSEM = pooled standard error of the mean. CG = confinement group (*n* = 31), PG = pasture group (*n* = 26). * = results highlighted with this symbol referring to week of paddock rotation. G = group, T = time, GxT = interaction of group and time. DMI = dry matter intake. TMR = total mixed ration; The CG stayed on a TMR-based diet during the entire trial, while the PG was slowly introduced to a pasture-based ration: week 0 and 1 = TMR, week 2 = TMR and 3 h pasture/day, week 3 and 4 = TMR and 12 h pasture/day, and week 5 to 11 = pasture and 4.5 kg DM concentrate cow/day.

**Table 4:** Effect of a ration change from an indoor-based total mixed ration (TMR) to pasture on blood cell count ^1.^

Variale ^1^	Group ^2^	Week												PSEM ^3^	*p*-Value		
		0	1	2	3	4	5	6	7	8	9	10	11		Group (G)	Time (T)	GxT
RBC	CG	5.59 ^a^	5.41 ^ab^	5.41 ^ab^	5.45 ^ab^	5.50 ^ab^	5.44 ^ab^	5.45 ^ab^	5.42 ^ab^	5.42 ^ab^	5.38 ^ab^	5.26 ^b^	5.24 ^b^	0.1	0.114	<0.001	<0.001
	PG	5.36 ^c^	5.36 ^c^	5.33 ^c^	5.54 ^bc^	5.73 ^ab^	5.71 ^ab^	5.87 ^a^	5.77 ^ab^	5.68 ^ab^	5.59 ^abc^	5.75 ^ab†^	5.54 ^bc^				
HGB	CG	8.59	8.36	8.33	8.23	8.62	8.66	8.62	8.43	8.41	8.29	8.22	8.28	0.16	<0.1	<0.001	<0.001
	PG	8.21 ^f^	8.28 ^ef^	8.45 ^def^	8.54 ^def^	9.20 ^abc^	9.30 ^ab^	9.23 ^abc^	9.15 ^abc^	8.83 ^bcd^	8.77 ^cde^	9.05 ^abc†^	9.37 ^a**^				
HCT	CG	25.0	24.2	24.2	24.3	24.6	24.4	24.4	24.3	24.5	24.3	23.7	23.8	0.5	<0.05	<0.001	<0.001
	PG	24.2 ^d^	24.3 ^cd^	24.2 ^d^	25.2 ^bcd^	26.2 ^ab^	26.3 ^ab^	26.7 ^a^	26.1 ^ab^	25.8 ^abc^	25.5 ^abcd^	26.0 ^ab†^	25.3 ^abcd^				
MCV	CG	44.7	44.8	44.8	44.8	44.9	44.8	44.9	44.8	45.2	45.2	45.1	45.4	0.7	0.534	<0.05	<0.1
	PG	45.4 ^b^	45.5 ^ab^	45.6 ^ab^	45.7 ^ab^	45.9 ^ab^	46.2 ^a^	45.6 ^ab^	45.5 ^ab^	45.6 ^ab^	45.8 ^ab^	45.4 ^ab^	45.8 ^ab^				
MCH	CG	15.4 ^cd^	15.5 ^bcd^	15.4 ^cd^	15.2 ^def^	15.7 ^abc^	15.9 ^a^	15.8 ^ab^	15.6 ^abc^	15.5 ^bcd^	15.4 ^cde^	15.6 ^abc^	15.8 ^ab^	0.3	0.432	<0.001	<0.001
	PG	15.4 ^e^	15.5 ^ef^	15.9 ^cd^	15.5 ^e^	16.1 ^bc^	16.3 ^b^	15.8 ^cde^	15.9 ^cd^	15.6 ^de^	15.7 ^de^	15.8 ^cde^	17.0 ^a^				
MCHC	CG	34.4 ^def^	34.6 ^cde^	34.5 ^cde^	33.8 ^f^	35.0 ^abc^	35.6 ^a^	35.3 ^ab^	34.7 ^bcd^	34.4 ^def^	34.1 ^ef^	34.7 ^cde^	34.3 ^def^	0.1	<0.05	<0.001	<0.001
	PG	33.9 ^f^	34.1 ^ef^	34.9 ^bc^	33.9 ^f^	35.2 ^bc^	35.4 ^b^	34.6 ^cde†^	35.0 ^bc^	34.3 ^def^	34.3 ^def^	34.8 ^cde^	36.9 ^a**^				
RDW	CG	16.0 ^e^	16.1 ^ef^	16.1 ^def^	16.3 ^cdef^	16.3 ^bcdef^	16.5 ^abcdef^	16.7 ^abcdef^	16.8 ^abcde^	16.8 ^abc^	16.8 ^abcd^	17.0 ^ab^	17.1 ^a^	0.2	0.986	<0.001	0.188
	PG	16.2 ^b^	16.2 ^ab^	16.5 ^ab^	16.6 ^ab^	16.6 ^ab^	16.8 ^a^	16.7 ^ab^	16.7 ^ab^	16.8 ^ab^	16.6 ^ab^	16.7 ^ab^	16.7 ^ab^				
PDW	CG	15.6 ^d^	15.6 ^d^	15.7 ^d^	15.6 ^def^	15.9 ^d^	15.9 ^d^	16.0 ^cde^	16.6 ^bcd^	18.5 ^a^	18.1 ^ab^	17.9 ^abc^	19.3 ^a^	0.4	<0.05	<0.001	<0.01
	PG	15.6 ^c^	15.9 ^c^	15.6 ^c^	15.6 ^cdef^	15.9 ^c^	16.2 ^c^	16.0 ^cde^	18.4 ^b^	19.6 ^ab^	20.4 ^a*^	18.4 ^b^	19.4 ^ab^				

a–f: Different letters indicate the difference between weeks within particular groups (*p* ≤0.05). ^1^ RBC = red blood cells (10 ³ /μL); HGB = hemoglobin (g/dL); HCT = hematocrit (%); MCV = mean corpuscular volume (fL); MCH = mean corpuscular hemoglobin (pg); MCHC = mean corpuscular hemoglobin concentrations (g/dL); RDW = red blood cell distribution width (%); PDW = platelet distribution width (%). ^2^ CG = confinement group (*n* = 21); PG = pasture group (*n* = 22). G = group, T = time, GxT = interaction of group and time; The CG stayed on a TMR-based diet during the entire trial, while the PG was slowly introduced to a pasture-based ration: week 0 and 1 = TMR, week 2 = TMR and 3 h pasture/day, week 3 and 4 = TMR and 12 h pasture/day, and week 5 to 11 = pasture and 4.5 kg of DM concentrate/cow/day. ³ PSEM = pooled standard error of the mean. † *p* ≤0.10; * *p* ≤0.05; ** *p* ≤ 0.01; for group comparisons per week.

**Table 5:** Effect of a ration change from an indoor-based total mixed ration (TMR) to pasture on blood clinical chemistry variables ^1^.

Variable ^1^	Group ^2^	Week			PSEM ^3^	*p*-Value		
		0	6	11		Group (G)	Time (T)	GxT
Albumin (g/L)	CG	36.6	36.6	36.6	0.6	0.172	0.785	0.697
	PG	35.4	35.9	35.9				
Total protein (g/L)	CG	68.3	70.1	70.1	1.3	<0.05	<0.05	0.553
	PG	68.1	71.0	72.5				
Cholesterol (mg/dL)	CG	222.8	211.5	201.0	7.6	<0.05	<0.05	0.133
	PG	212.2	214.7	206.1				
AST (IU/L)	CG	82.8	77.3	74.8	5.3	0.381	0.355	0.257
	PG	84.5	81.4	86.3				
γ-GT (IU/L)	CG	42.2	39.6	39.4	3.7	0.285	0.285	0.494
	PG	43.9	36.4	44.0				
Bilirubin (mg/dL)	CG	0.37	0.36	0.35	0.01	<0.01	<0.01	<0.001
	PG	0.37 ^b^	0.42 ^a**^	0.36 ^b^				
GLDH (IU/L)	CG	35.8	31.7	30.6	3.4	0.139	0.139	0.500
	PG	32.9	28.1	37.0				

^a,b^ Different letters indicates difference between weeks within particular groups (*p* ≤ 0.05). ^1^ γ-GT = γ-glutamyltransferase; GLDH = glutamate dehydrogenase. ^2^ CG = confinement group (*n* = 21); PG = pasture group (*n* = 22). G = group, T = time, GxT = interaction of group and time; The CG stayed on a TMR-based diet during the entire trial, while the PG was slowly introduced to a pasture-based ration: week 0 and 1 = TMR, week 2 = TMR and 3 h pasture/day, week 3 and 4 = TMR and 12 h pasture/day, and week 5 to 11 = pasture and 4.5 kg DM concentrate cow/day. ^3^ PSEM = pooled standard error of the mean. ** *p* ≤ 0.01 for group comparisons per week.

## Appendix A

**Table A1:** Pasture plant diversity.

Plant species (%)	Paddock 1 *	Paddock 2 *	Paddock 3 *	Paddock 4 *
Grass	80.8 ± 4.4	71.2 ± 32.6	81.3 ± 16.3	92
Herbs	5.8 ± 4.6	8.3 ± 6.7	11.3 ± 6.5	2.0
Legumes	1.8 ± 3.0	6.7 ± 8.3	5.0 ± 8.7	6.0
Lolium perenne	42.3 ± 34.5	27.2 ± 11.2	32.8 ± 18.8	40
Phleum pretense	32.3 ± 31.7	28.2 ± 10.6	22.5 ± 31.7	10
Poa trivalis		7.0 ± 0.0	2.5 ± 0.7	
Poa annua		4.5 ± 2.5		
Holus Lanatus			5.0 ± 0.0	
Dactylis glomerata	2.0 ± 0.5	14.2 ± 10.8	5.0 ± 0.0	
Poa pratensis	5.0 ± 2.4	5.0 ± 0.0	6.5 ± 2.1	7.0
Elymus repens	0.7 ± 1.2			
Festuca pratensis		5.0 ± 0.0	72.0 ± 0.0	35
Festuca rubra			4.0 ± 0.0	
Stellaria media	5.3 ± 4.6	3.3 ± 2.0	9.0 ± 5.2	
Achillea millefolium			5.0 ± 0.0	
Lamium Purpureum		0.5 ± 0.0		
Capsella bursa-pastoris		2.0 ± 1.0		
Taracum officinale			4.0 ± 0.0	1.0
Veronica chamaedrys				
Geranium dissectum		10.0 ± 0.0		
Trifolium repens	2.3 ± 4.0	7.3 ± 9 0	20.0 ± 0.0	
Trifolium pratense				6.0

Four samples (1 m × 1 m) per paddock were taken, with exception of paddock 4 (only one sample) for characterization of plant species. * The rest up to 100% was nonvegetated.
